# Too Much Compromise in Today's CRISPR Pipelines

**DOI:** 10.1089/crispr.2019.0015

**Published:** 2019-06-21

**Authors:** Richard Fox

**Affiliations:** Data Science, Inscripta, Inc., Boulder, Colorado.

## Abstract

CRISPR-based editing is a revolutionary tool for genome engineering and discovery. The pace of innovation in this young field is truly astonishing, and yet significant gaps remain in the set of capabilities offered. In particular, scalable (massively parallel and multiplex) editing is only available for a small variety of edit types, placing fundamental limits on the kinds of studies researchers can perform. Additional advancements are needed to realize the full potential of the technology.

## Introduction

Describing the adoption of CRISPR technology for genome editing without sounding hyperbolic is virtually impossible. Those who have been in the life sciences field long enough to remember the early days of next-generation sequencing, an advance that gained widespread traction with remarkable speed, still see the pace of CRISPR adoption as unprecedented.

When a technique becomes so popular so quickly, it is tempting to get swept up in the enthusiasm and avoid questioning its limitations. But even as the excitement around CRISPR continues unabated, it is important for the community to recognize that current gene-editing pipelines face serious technical and other challenges. Chief among them is scalability. CRISPR cannot realize its full potential for applications in health care and industrial and agricultural biotechnology without a transformation in the throughput at which these experiments can be done. Other challenges include expanding the variety of edit types that can be made, performing those edits in combination, and tracking all edits for robust analysis and optimal learning.

Scaling up the throughput of CRISPR experiments is critical, as is expanding the variety of edit types and combinations of edits that can be performed. There is great interest in introducing larger inserts or swapping out promoters, transcription factor binding sites, and other elements. Unfortunately, there are no robust, scalable approaches for making these kinds of edits today. For example, while base editors are a useful addition to the toolbox of genome engineering and discovery, the technology is fundamentally limited because of the low variety of edit types available to researchers.

Another much-needed advance is in the space of combinatorial editing—the ability to create combinations of mutations in the same genome. The effort needed to carry this out today, even at the lowest throughput, is prohibitive for most labs, particularly when the target sites are spread throughout the genome and are beyond the scope of single base edits. However, the approach is essential for building sequence-to-function relationships that can be used to engineer biological systems rapidly.^1^

Today's CRISPR pipelines require too much compromise. Scientists who would ideally like to test many edits (both individually and in combination) must instead choose a small number of edits because it is not currently feasible—technically or financially—to study more. For example, industrial biotechnologists looking to engineer a high-performing microbial strain for a desired phenotype must limit the number of strains they can create and evaluate. And researchers who are not limited by models or a small number of preconceived ideas to test and who are looking to edit genomes on a massive scale find themselves designing far fewer audacious experiments for the sake of practicality.

There is no question that CRISPR is a game-changing approach to biological engineering, and it is important to acknowledge that CRISPR is still in its infancy. It has come a long way in the short six years since it was first discovered, and for many applications there is simply no other technology that can compete with CRISPR's current capabilities in terms of scale, cost, and precision. So, while we should be excited as a community to have this amazing tool, we will never accomplish all that CRISPR promises without addressing its limitations. Only then can we focus on the technological and other innovations that will be required to make the most of this exquisite tool.

## Current Limitations

The most pressing challenge in CRISPR workflows today is the lack of scalability. For applications in genome engineering and discovery, there are often virtually unlimited possibilities to test. Instead, we down-select because there is no reasonable way to intervene at thousands or tens of thousands of target loci in a genome beyond simple knockout screens^2^ or limited-variety base editors.^3^ The community has made tremendous progress at multiplexed genome editing through base editing^4^ and gene disruption^5^ where dozens to as many as 10,000 or more edits have been made to a single genome. These advancements are akin to having a word processor capable of making single-letter changes or randomly deleting a few letters at many locations. What the field needs now is a fully featured editing program capable of modifying entire words, sentences, and paragraphs at many locations.

For example, in genome discovery and engineering applications, suppose you gathered a large number of genetic differences of varying sizes between cell lines from historical studies and wanted to recapitulate them in a clean background to determine which ones were causal or to study how they function. While comparative genomics and adaptive evolution databases may contain many thousands of candidates to evaluate, it is not currently feasible generally to test more than a few dozen, or perhaps a few hundred of these in the most well-provisioned groups.

More broadly, successful outcomes in metabolic engineering depend on the ability to target many proteins in a metabolic pathway as well as numerous loci throughout the genome in a generalized manner across a wide variety of edit types and sizes. What scientists need is to be able to make changes at any and all regions in the genome that might be relevant for a phenotype of interest, such as improved enzyme or regulatory function. Today, however, they are restricted to altering just a few loci in precise ways.^6^

Beyond the editing process itself, improvements are needed for surrounding elements in the CRISPR ecosystem. Many scientists struggle with design steps for guide RNAs, cassettes, and nucleases. Developing more robust, turnkey alternatives that automate every step of the process, from design to manufacture of reagents and cell lines, would allow researchers to focus on their science instead of on the CRISPR workflow. In addition, trackability (the ability to read back out what you wrote) is increasingly important, especially as editing experiments become more complex. Large numbers of combinatorial changes, for instance, may not allow scientists to use plasmid handles to track changes. For optimal CRISPR results and the ability to iterate on previous cycles, users must have a clear and reliable record of all changes made.

## Key Applications

**FIG. 1.**
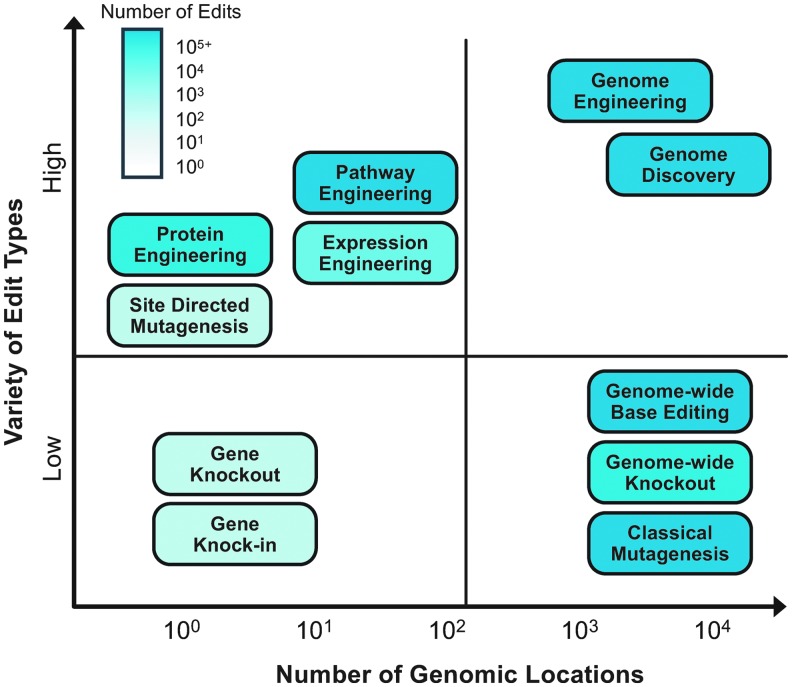
Key features of editing applications and techniques. The *x*-axis shows the number of genomic loci targeted by different methods and applications. The *y*-axis represents the variety of edit types (insertions, deletions, and swaps of varying sizes and combinations thereof) made possible by the technique or required by an application. The applications and techniques are colored in the background to represent a high (cyan) or low (white) number of edits.

There are many applications that would benefit tremendously from more robust, scalable CRISPR techniques (Fig. 1). Genome engineering, for example, could flourish if it were possible to perform more edits and more types of edits in combination. This would be transformative for R&D groups of all sizes, from small biotechnology companies and academic labs to the large biological foundries that have been established to create biosystems to produce fine and commodity chemicals, peptides, and proteins efficiently.^7^

Scaling CRISPR would also enable a leap forward in our ability to understand the rules of biology as a foundation for future engineering efforts. Despite decades of research in yeast and *Escherichia coli*, for instance, we still cannot adequately explain the function and activity of many genetic components.^8^ Such genome discovery applications will allow researchers to elucidate the role of regulatory regions or proteins (and their combinations) and will require in-depth investigations of genotypic and phenotypic changes that result from extensive editing experiments.

## Looking Ahead

Genome biology has a long history as an observational science, but thanks to CRISPR, it is now becoming much more of an interventional science. Intervening at the genomic level and then studying the broader results of those interventions are critical steps in establishing causality and understanding how a system works. The ability to elucidate cause and effect is the gold standard for mastering the rules governing how any system, biological or otherwise, truly operates and being able to predict (and prescribe) its performance.^9^

We will not achieve that level of understanding without being able to perturb biological systems at all levels, from effecting small base-pair changes to swapping in or out or modifying larger segments such as promoters, transcription factors, and other regulatory elements. It's the only way to decode the web of interactions that direct biology and reveal the full nature of the system.

We must not accept CRISPR as it is today, but rather continue pushing against the current limitations.^10,11^ When a tool is this powerful and has so much potential, it is our responsibility to ensure that it can be used as broadly as possible for all constructive applications. As a community, we must work together to realize the full promise of CRISPR, genome biology, and engineering.
